# Psychological Vulnerability of French University Students during the COVID-19 Pandemic: A Four-Wave Longitudinal Survey

**DOI:** 10.3390/ijerph18189699

**Published:** 2021-09-15

**Authors:** Elodie Charbonnier, Sarah Le Vigouroux, Aurelie Goncalves

**Affiliations:** UNIV. NIMES, APSY-V, F-30021 Nîmes Cedex 1, France; sarah.le_vigouroux_nicolas@unimes.fr (S.L.V.); aurelie.goncalves@unimes.fr (A.G.)

**Keywords:** COVID, anxiety, depression, coping, university, lockdowns

## Abstract

Background: Many studies have highlighted the negative mental health consequences of lockdowns. However, to date, we do not know how these consequences change over time. The first objective of the present study was to track changes in adjustment strategies and clinical issues among French university students at different times of the pandemic. The second objective was to investigate the psychological and situational factors contributing to students’ anxiety and depressive symptoms. Method: This cohort study was conducted between 23 April and 11 December 2020. Measurements were performed four times: during France’s first national lockdown, during the period after lockdown, when universities were open, and finally during the second national lockdown. A total of 1294 university students were initially included, and 91 students completed the four measurement points over a 7-month period. Coping strategies (with the Brief-COPE), health concerns (with two questions), anxiety and depressive symptoms (with the HADS) were measured. Results: Results showed an evolution over time of anxiety (χ^2^ = 21.59 ***) and depressive (χ^2^ = 29.73 ***) symptoms. Depressive symptoms are significantly higher during lockdown periods compared to unlockdown periods. Anxiety symptoms are likewise particularly high during the two lockdowns, but also when the universities reopen. At different times, anxiety and depressive symptoms were positively associated with maladaptive strategies, such as the self-blame (rho between 0.33 and 0.51) and negatively with adaptive strategies, such as the positive reframing (rho between −0.23 and −0.44). Conclusions: The trajectory of anxiety, which is elevated even in the absence of lockdown, raises concerns about the long-term effects of the pandemic on these symptoms.

## 1. Introduction

On 11 March 2020, the World Health Organization declared SARS-CoV-2 to be a global pandemic [1]. In France, all universities closed on 16 March 2020 [2]. In September 2020, face-to-face teaching resumed in French universities, but with new constraints (e.g., fewer students in classrooms, mask wearing) and major changes in teaching (e.g., distance and/or hybrid education). In October 2020, several French universities closed again, owing to high infection rates among students. On 30 October 2020, the French Government imposed a second national lockdown and all universities had to close again [3]. The lockdown ended on 15 December 2020, but universities remained closed to students, except for a few courses involving practical work [4]. Face-to-face teaching was partially (approximately 20%) resumed in February 2021 [5].

Since the beginning of the pandemic, several authors [6,7,8] have highlighted the various challenges faced by university students (e.g., widespread transition to remote online learning, changes in assessment and examinations), as well as the negative impact on their mental health. In France, a clear deterioration in their mental health has been observed, with very high levels of anxiety and depressive symptoms, particularly during the first lockdown [9,10,11,12,13]. This can partly be explained by the fact that people who were already experiencing high levels of psychological distress prior to the pandemic have been most vulnerable to the effects of lockdown [14,15], and university students are known to be subject to psychological distress (for reviews, see [16,17]). In addition, situational factors related to COVID (e.g., loss of employment and financial stressor, increased domestic violence, intensive exposure to hopelessness stories by the media) play an important role in understanding the mental health effects of the pandemic, as indicated in the article of [18].

Similarly, more specifically among students, the role of different situational factors, such as social media exposure [19], dorm closures and relocations, distance from relatives or university [6,7] and, more broadly, loneliness and isolation [20,21,22,23], have been highlighted.

Before COVID-19, a key feature of students’ psychological distress was difficulty coping with an accumulation of hassles, such as academic pressure, schedule changes, financial difficulties, and even isolation [24]. The pandemic has exposed students to new and unprecedented events (e.g., switch to online learning, online examinations, regular and substantial changes to their schedule) that challenge their coping strategies [23,25]. *Coping strategies* can be defined as cognitive and behavioral efforts undertaken by individuals to deal with stressful situations [26]. They can be categorized as maladaptive or adaptive. *Maladaptive strategies* (e.g., behavioral disengagement) refer to rigid and maladaptive behaviors that do not improve the situation and may increase stress. Conversely, *adaptive strategies* (e.g., acceptance) refer to efforts that promote resolution and reduce stress [27]. Recent research has shown that the more university students resort to avoidance strategies during lockdowns, the higher their levels of anxiety and depressive symptoms [12,28]. Thus, students’ psychological status may also depend on individual factors, particularly the coping strategies they use to deal with the pandemic.

In sum, the pandemic has clearly had a major impact on the mental health of university students, as evidenced by their high levels of anxiety and depressive symptoms. Almost all studies drew this conclusion for the first lockdown, but we have no knowledge about how students’ mental health has fared since then. Authors suggest that the psychological effects will persist long after COVID-19 has peaked [21,29,30,31], but we have no data to confirm this. Therefore, the first objective of the present study was to track changes in clinical issues and adjustment among French university students over time. More specifically, our study proposes to describe the trajectory of coping strategies, anxiety and depression symptoms in French university students during different phases of the COVID-19 pandemic (during two periods of lockdown and two periods after lockdown). The second objective was to investigate the psychological and situational factors contributing to students’ anxiety and depressive symptoms.

Concerning our first objective, we hypothesized that during lockdowns, compared with periods after lockdown.

**Hypothesis 1** **(H1).** *University students exhibit more severe anxiety and depressive symptoms.*

**Hypothesis 2** **(H2).** *University students are more concerned about health.*

**Hypothesis 3** **(H3).** *University students use more maladaptive strategies (e.g., behavioral disengagement) and fewer adaptive strategies (e.g., acceptance).*

Concerning our second objective, we hypothesized that the higher their levels of anxiety and depressive symptoms.

**Hypothesis 4** **(H4).** *The more concerned university students are about health.*

**Hypothesis 5** **(H5).** *The more university students use maladaptive strategies and the less they use adaptive strategies.*

## 2. Methods

### 2.1. Participants

Initially, a total of 1294 university students were assessed (Time 1). At each time point, all participants who had responded at Time 1 (even if they had not responded at Time 2 or Time 3) were re-solicited. Thus, at Time 2, 373 responded, at time 3, 284 responded and at Time 4, 160 responded. The characteristics of the samples at each measurement time are presented in Table 1. Finally, 91 students completed all four measurement points over a 7-month period, their characteristics are detailed in the results section.

### 2.2. Instruments

Anxiety and depressive symptoms were assessed using a French version of the Hospital Anxiety and Depression Scale (HADS [32]). This 14-item self-report questionnaire assesses the intensity of both anxiety (7 items) and depressive symptoms (with 7 items) during the previous week. Scores range from 0 to 21 for each dimension. The higher the score, the more intense the anxiety or depressive symptoms (a score ≤ 7 means no symptoms, a score of 8–10 means possible symptoms, and a score ≥ 11 means probable symptoms). Although this scale has not been specifically validated with students, it is used in many epidemiological studies in the general population to identify the existence of a symptomatology and to assess its severity.

Coping strategies were assessed using a French version of the situational version of the Brief-COPE [27]. This self-report scale assesses 14 coping strategies (2 items per strategy): nine adaptive strategies (active coping, planning, instrumental support, use of emotional support, venting, positive reframing, humor, acceptance and religion), and five maladaptive strategies (behavioral disengagement, self-distraction, self-blame, denial, and substance use). Participants rated each of the 28 items on a 4-point Likert scale ranging from Never to Always. At Times 1 and 4, they were instructed to refer to a stressful situation related to the lockdown. At Times 2 and 3, they were asked to refer to a stressful situation related to the pandemic. Higher scores reflected higher levels of strategy use. The French validation of this scale was performed with French students and has good psychometric properties.

We considered two situational factors: participants’ level of concern about their own health and their level of concern about their relatives’ health with regard to the COVID-19 pandemic (two scales ranging from 0 to 100).

### 2.3. Procedure

Data were collected anonymously at four timepoints between 23 April and 11 December 2020, via an online survey designed with Qualtrics software (Qualtrics, Provo, UT, USA). To track changes in the students’ psychological state, we conducted measurements at four timepoints: (1) during France’s first national lockdown (between 23 April and 8 May); (2) during the period after lockdown when universities remained closed and the summer vacation had begun (9–23 June); (3) when universities were open (12–23 October); and (4) during the second lockdown (between 20 November and 11 December). For the first time (Time 1), a link to the survey was sent by e-mail to teachers in several faculties (i.e., Science, Psychology), at various French universities (i.e., Nîmes, Lorraine), and was also distributed via students’ social media (e.g., Facebook groups). Our only criterion for inclusion was to be a student at a French university. Participants agreed to participate in this study after reading a consent form. They were informed that their participation was voluntary, and they could withdraw at any time. No personal data allowing the identification of the participants were collected, except their email addresses. These data were separated from the other data and exclusively used to send invitations to participants at each measurement time. All the procedures contributing to this work were undertaken in compliance with the ethical standards of the relevant national and institutional committees on human experimentation and with the 1975 Declaration of Helsinki, revised in 2008.

### 2.4. Statistical Analyses

First, we studied the nature of distribution of our variable with the Shapiro–Wilk test. Since the results (Table 2) indicate distributions that do not follow the normal distribution, we performed non-parametric tests afterwards. Second, to study the effect of time on anxiety, depression, coping and health concerns, repeated measures ANOVAs with a Friedman test were performed. Theses analyses were conducted only on participants who responded to all measurement times. Third, to analyze the associations between coping strategies, health concerns and symptoms, we ran Spearman correlations analyses for each timepoint. Since our data do not follow the normal distribution, only non-parametric tests were performed.

## 3. Results

In order to accomplish the first objective of this research, which was to track changes in adjustment strategies and clinical issues, only participants who responded to the four measurement times were included (N = 91). This sample comprised 91 students (73.62% female, M_age_ = 22.35, SD = 5.84), from different French universities (50.54% Nîmes, 28.57% Lorraine, 12.08% Strasbourg and 8.79% other universities). These were primarily undergraduate students (32.97% in first years, 29.67% in second years and 24.18% in third years), 10.99% were master’s students and 2% doctoral students. In line with our first hypothesis, results indicated that levels of anxiety and depressive symptoms were high during the two lockdowns (Times 1 and 4; Table 2). Indeed, repeated measures ANOVA (Table 2 and Table 3) showed differences between our four measurement times in depressive symptoms (η^2^ = 0.15) and, to a lesser extent, in anxiety (η^2^ = 0.09). As expected, it was during the initial period after lockdown (Time 2), when universities were closed and the summer vacation had begun, that the decrease in symptoms was most pronounced. It is important to note that differences between anxiety and depressive symptoms emerged at Time 3 (i.e., when universities reopened). Anxiety rose again during this period, and remained high during the subsequent second lockdown (Time 4). By contrast, depressive symptoms remained low at Time 3 but increased again during the second lockdown (Time 4). In other words, depressive symptoms are significantly higher during lockdown periods (Times 1 and 4) compared to unlockdown periods (Times 2 and 3).

Furthermore, in line with our second hypothesis, our results indicate time differences in students’ concerns about their relatives’ health (η^2^ = 0.19) and, to a lesser degree, about their own health (η^2^ = 0.02, Table 2 and Table 3). The evolution of their concern about the health of their relatives is quite similar to that of anxiety. In other words, university students were more concerned during the first lockdown (Time 1) than during the period just after lockdown (Time 2), their concern rose again when universities reopened (Time 3), and remains high during the subsequent second lockdown (Time 4). In the same line, their concerns about their own health increase in Time 3 and remain high in Time 4, compared to the summer period (Time 2).

Finally, concerning coping strategies, we observed slight variations in coping strategies over time (Table 2 and Table 3), but the effects were very small (η^2^ included between 0.01 and 0.05. Furthermore, the evolution of strategies is weakly dependent on the alternation between lockdown and unlockdown, contrary to our hypotheses. More precisely, they use more planning (adaptive strategy) during periods of lockdown (Times 1 and 4). In addition, compared to the first lockdown (Time 1), they use denial less once the university reopens (Time 3) as well as during the second lockdown (Time 4). Finally, they use acceptance (adaptive strategy) less during the reopening of the university (Time 3) than during previous times (Times 1 and 2).

In order to accomplish the second objective of this research, which was to investigate factors related to anxiety and depressive symptoms during the pandemic, all participants were included (N_T1_ = 1294; M_age_ = 21.28 years ± 4.73; n_T2_ = 373; M_age_ = 22.12 years ± 5.70 n_T3_ = 284; M_age_ = 21.95 years ± 5.33; n_T4_ = 160; M_age_ = 21.81 years ± 6.05). Participants’ characteristics at the four timepoints are set out in Table 1. In accordance with our fourth hypothesis, anxiety was positively and moderately associated with health concerns. Similarly, depressive symptoms were weakly but positively associated with health concerns (Table 4). Furthermore, in line with our fifth hypothesis, at different times of the COVID-19 pandemic, anxiety and depressive symptoms were positively associated with maladaptive strategies, such as behavioral disengagement, denial, substance use or self-blame. Furthermore, anxiety and depressive symptoms were negatively associated with adaptive strategies, such as acceptance, humor or positive reframing (Table 4).

## 4. Discussion

The COVID-19 pandemic has had a major impact on higher education and imposed new constraints (e.g., smaller numbers in the classroom, distance and/or hybrid education, distance evaluation) on university students. High levels of anxiety and depressive symptoms among university students were reported during the first lockdown in France [10,12], as well as on an international level [9,11,13]. However, we still have only limited knowledge about how these symptoms have changed since then. The first objective of the present study was therefore to describe the trajectories of coping strategies, anxiety and depressive symptoms among French university students, by conducting measures at different timepoints (two lockdowns and twice during the intervening period). The second objective was to investigate the role of coping strategies and health concerns on anxiety and depressive symptoms.

First, results indicated that levels of depressive symptoms among French university students were particularly high during the two lockdowns, with nearly 30 and 37% reporting possible symptoms. These results are consistent with general trends observed in previous studies conducted among students during the first lockdown [10,13,33]. Our study is the first, to our knowledge, to report rates for the second lockdown in France. More interestingly, we observed a significant decrease in depressive symptoms just after the first lockdown, which remained low until the second lockdown, at a rate of 14–16%, compared with the pre-COVID rate of 30% [34]. The trajectory of depressive symptoms suggests that the COVID-19 pandemic may not have a major long-term effect on students’ depressive symptoms, contrary to findings for previous pandemics (e.g., [35,36,37]). This may in part be explained by the age of our participants. Indeed, young adults have two primary social goals: social acceptance and autonomy, with friends as preferred partners [38]. During lockdown, these goals and social partners may have been impeded, which may have contributed to depressive symptoms’ increase. Conversely, once lockdown ended, these goals and partners may have been restored, which may partially explain the decrease in their symptoms.

High levels of anxiety were also exhibited by university students during the two lockdowns, with a rate of possible anxiety symptoms of 45 to 48%. This rate fell substantially just after the end of the first lockdown, but increased again at the start of the academic year and the resumption of face-to-face teaching, with a rate of 46% of probable anxiety, compared with the pre-COVID rate of 24.2% [39]. The start of the academic year can be a stressful time for students, and it may have been exacerbated by the constraints imposed by COVID-19 (e.g., hybrid education, mask wearing), and by potential obstacles to distance learning (e.g., technological, personal, family [40]). The trajectory of anxiety suggests that (1) students’ anxiety remained particularly high during the pandemic, even in the absence of lockdown, and (2) it is difficult to distinguish between the effects of lockdown and the effects of academic constraints, whether or not these are related to COVID-19. It is probably the accumulation of these factors that contributed to students’ anxiety, and this should be a major concern for universities.

Concerning contributing factors, results indicated that students’ anxiety and depressive symptoms were positively associated with health concerns and maladaptive strategies (e.g., behavioral disengagement, self-blame, denial). The associations between health concerns, anxiety and depressive symptoms can be explained by the many uncertainties associated with the virus and its spread. In France, as in many other countries around the world, the pandemic fueled contradictory reporting and intense controversy in the media [41]. Satici et al. [42] showed that an inability to tolerate uncertainty can precipitate fear of the virus and impact negatively on psychological wellbeing. Associations between maladaptive strategies and anxiety and depressive symptoms had been established prior to the pandemic [43,44], and were confirmed during the first lockdown [28]. However, our study is the first to highlight changes in these strategies during the pandemic. Dawson and Golijani-Moghaddam [28] concluded that the isolating and restrictive context of lockdown may prevent university students from tapping into their usual repertoire of coping strategies. On the contrary, our results suggest that students did use strategies, and more particularly strategies that were adapted to the situation (i.e., acceptance and positive reframing), but were nonetheless unable to effectively regulate their psychological distress. This result is consistent with studies of age-related differences in emotion regulation showing that the effectiveness of strategies improves with age [45].

In sum, this research is the first to report changes in anxiety and depressive symptoms among university students at different times during the pandemic. Finally, to better understand the fluctuations of anxiety and depressive symptoms over time, future studies should further explore factors that may vary between periods of lockdown and easing of restrictions. For example, housing characteristics (e.g., poor housing, poor views and scarce indoor quality) can be detrimental to student’s mental health [46], and lockdown makes it even more unbearable, especially since it limits the opportunities for outdoor activities [41]. Furthermore, social support appeared to have been a protective factor against stress for French university students during lockdown [47], as the latter increases loneliness and isolation [20,21]. Finally, the role of certain dispositional factors, such as personality traits, which are strongly associated with psychopathology [48,49,50,51], would also merit further study in the specific context of the COVID-19 pandemic.

The present study had several limitations, meaning that some results should be interpreted with caution. First, regarding the representativeness of our sample, we were unable to include newly enrolled (i.e., as of September 2020) students, our sample was predominantly female, and the participants come mainly from three universities. These elements lead us to express reserves about the generalization of our result. Furthermore, although our initial sample size was very large, it decreased substantially thereafter. In addition, we have many participants who did not respond to at least one measurement time, which forced us to exclude them from longitudinal analyses. Nevertheless, loss to follow-up is quite common in longitudinal studies. Second, Time 2 coincided with the start of the summer vacation, making it difficult to dissociate the effects of unlockdown from those of the vacation.

## 5. Conclusions

The present study revealed a particularly high prevalence of anxiety and depressive symptoms during the two lockdowns. Nevertheless, differences in the development of anxiety and depressive symptoms emerge after the first lockdown. More precisely, student anxiety is high during the reopening of the university as during lockdowns. This contrasts with the trajectory of depressive symptoms that increases exclusively during lockdowns. These results may raise some concerns about the long-term effects of the pandemic on students’ anxiety symptoms. However, they also allow for more optimism that the pandemic may not have a lasting impact on students’ depressive symptoms. There are a number of limitations to this study, including the representativeness of our sample, that lead to caution in considering the results. Replications need to be conducted with a more representative population before one may be able to generalize such findings.

## Figures and Tables

**Table 1:** Characteristics (numbers and percentages) of all respondents at each measurement time.

Characteristics	T1 (N = 1294)	T2 (n = 373)	T3 (n = 284)	T4 (n = 160)
	Number (%)	Number (%)	Number (%)	Number (%)
**Gender**				
Female	1006 (77.7)	310 (83.1)	232 (81.7)	124 (77.5)
Male	268 (20.7)	54 (14.5)	44 (15.5)	29 (18.1)
Other	20 (1.6)	9 (2.4)	8 (2.8)	7 (4.4)
**University**				
Nîmes	558 (43.12)	179 (47.99)	142 (50)	78 (48.75)
Lorraine	370 (28.59)	82 (22.25)	70 (24.65)	47 (29.38)
Strasbourg	212 (16.38)	64 (17.16)	39 (13.73)	23 (14.38)
UCO Angers	86 (6.65)	20 (5.36)	12 (4.23)	5 (3.13)
Other	68 (5.26)	29 (7.77)	21 (7.39)	7 (4.38)
**Education Level**				
Undergraduate				
First year	486 (37.56)	100 (26.81)	96 (33.80)	57 (35.63)
Second year	314 (24.27)	106 (28.42)	72 (25.35)	47 (29.38)
Third year	323 (24.96)	98 (26.27)	69 (24.30)	35 (21.88)
Master’s				
Fourth year	82 (6.34)	34 (9.12)	27 (24.30)	15 (9.38)
Fifth year	74 (5.72)	26 (6.97)	13 (4.58)	3 (1.88)
PhD	11 (0.85)	8 (2.14)	6 (2.11)	3 (1.88)
Undefined	4 (0.31)	2 (0.54)	1 (0.35)	0

**Table 2:** Comparison of coping strategies, health concerns and anxiety–depressive symptoms scores of French students at different times during the COVID-19 pandemic (two periods of lockdown and two unlockdown periods) with Friedman test of non-parametric ANOVA (N = 91).

	Min-Max	Shapiro-Wilk		T1			T2			T3			T4		χ^2^
			M	SD	Mdn	M	SD	Mdn	M	SD	Mdn	M	SD	Mdn	
Concern about own health	0–100	0.90 ***	27.03	25.14	20	22.73	22.42	17	28.41	23.43	21	27.73	25.45	20	7.90 *
Concern about relatives’ health	0–100	0.94 ***	60.31	28.99	65	40.97	28.3	40	59.34	27.82	62	56.42	28.92	60	47.54 ***
Anxiety symptoms	1–21	0.96 **	7.97	4.14	7	6.46	4.12	6	7.60	4.12	7	8.29	4.57	7	21.59 ***
Depressive symptoms	0–18	0.96 ***	6.41	3.98	6	4.23	3.7	3	4.31	3.51	3	5.59	4.05	5	29.73 ***
Coping strategies															
Active coping	2–8	0.89 ***	3.68	1.44	3	3.69	1.74	4	3.76	1.44	4	3.83	1.53	4	0.98
Planning	2–8	0.91 ***	4.48	1.90	4	4.15	2.03	4	3.90	1.64	4	4.48	1.8	4	12.06 **
Using instrumental support	2–8	0.87 ***	3.69	1.65	3	3.64	1.95	3	3.82	1.49	4	3.64	1.41	4	1.09
Using emotional support	2–8	0.87 ***	3.99	1.88	4	3.99	2.03	4	3.85	1.6	4	4.14	1.81	4	4.09
Venting	2–8	0.91 ***	4.29	1.73	4	4.24	2.06	4	4.39	1.52	4	4.38	1.5	4	0.23
Positive reframing	2–8	0.94 ***	5.32	1.68	5	5.07	2.08	5	4.89	1.55	5	4.99	1.75	5	5.23
Humor	2–8	0.88 ***	4	1.79	4	3.67	2.03	4	3.72	1.71	4	3.84	1.87	4	3.56
Acceptance	2–8	0.90 ***	6.25	1.47	6	6.16	2.14	7	5.94	1.51	6	6.13	1.45	6	12.20 **
Religion	2–8	0.56 ***	2.69	1.38	2	2.63	1.51	2	2.67	1.22	2	2.58	1.14	2	1.43
Behavioral disengagement	2–8	0.89 ***	3.71	1.55	3	3.33	1.63	3	3.61	1.59	3	3.95	1.69	4	7.49
Self-distraction	2–8	0.95 ***	4.89	1.60	5	4.70	1.89	5	4.60	1.39	5	4.64	1.51	5	3.30
Self-blame	2–8	0.83 ***	3.66	1.66	3	3.30	1.62	3	3.56	1.53	3	3.77	1.67	3	2.56
Denial	2–8	0.73 ***	2.87	1.19	2	2.64	1.44	2	2.59	1.16	2	2.52	1.03	2	9.53 *
Substance use	2–8	0.47 ***	2.59	1.42	2	2.40	1.27	2	2.38	1.06	2	2.69	1.60	2	1.40
**Frequencies**			n	(%)		n	(%)		n	(%)		n	(%)		
None anxiety (≤7)			47	51.6		62	68.1		49	53.8		50	54.9		
Possible anxiety (8–10)			18	19.8		15	16.5		22	24.2		11	12.1		
Probable anxiety (≥11)			26	28.6		14	15.4		20	22		30	33		
None depression (≤7)			57	62.6		78	85.7		76	83.5		65	71.4		
Possible depression (8–10)			19	20.9		7	7.69		10	11		14	15.4		
Probable depression (≥11)			15	16.5		6	6.59		5	5.49		12	13.2		

Note: M, mean; SD, standard deviation; Mdn, median; * *p* < 0.05, ** *p* < 0.01 and *** *p* < 0.001.

**Table 3:** Comparisons of coping strategies, health concerns and anxiety–depressive symptoms scores of French students between each measurement time with Conover’s Post-Hoc test (N = 91).

	T1-T2	T1-T3	T1-T4	T2-T3	T2-T4	T3-T4
Concern about own health	1.21	1.42	0.88	2.63 **	2.08 *	0.54
Concern about relatives’ health	5.85 ***	0.18	1.45	6.04 ***	4.41 ***	1.63
Anxiety symptoms	3.47 ***	0.27	0.87	3.20 **	4.33 ***	1.14
Depressive symptoms	4.21 ***	4.72 ***	1.60	0.52	2.60 *	3.12 **
Coping strategies						
Planning	0.67	2.89 **	0.20	2.22 *	0.87	3.09 **
Acceptance	1.16	2.09 *	1.27	3.25 **	2.43 *	0.82
Denial	1.42	2.09 *	3 **	0.67	1.58	0.92

* *p* < 0.05, ** *p* < 0.01, and *** *p* < 0.001.

**Table 4:** Spearman correlations between anxiety and depressive symptoms, concerns about health (own and relatives’) and coping strategies at each timepoint.

	T1 (N = 1294)				T2 (n = 373)				T3 (n = 284)				T4 (n = 160)			
	Anxiety Symptoms		Depressive Symptoms		Anxiety Symptoms		Depressive Symptoms		Anxiety Symptoms		Depressive Symptoms		Anxiety Symptoms		Depressive Symptoms	
Concern about their health	0.37	***	0.15	***	0.36	***	0.22	***	0.39	***	0.18	***	0.26	***	0.14	
Concern about their relatives’ health	0.35	***	0.15	**	0.33	***	0.22	***	0.38	***	0.22	***	0.26	***	0.17	*
Coping Strategies																
Active coping	−0.04		−0.24	***	−0.03		−0.14	**	0.10		−0.20	**	0.03		−0.16	
Planning	−0.02		−0.19	***	0.002		−0.09		0.09		−0.10		0.04		−0.14	
Using instrumental support	0.28	***	0.06		0.19	***	−0.03		0.30	***	0.08		0.38	***	0.17	*
Using emotional support	0.35	***	0.15	**	0.24	***	0.11	*	0.36	***	0.10		0.44	***	0.20	*
Venting	0.08		−0.09	*	0.05		−0.08		0.13	*	−0.06		0.12		0.01	
Positive reframing	−0.36	***	−0.44	***	−0.25	***	−0.31	***	−0.23	***	−0.33	***	−0.23	**	−0.34	***
Humor	−0.33	***	−0.25	***	−0.19	***	−0.11	*	−0.23	***	−0.18	**	−0.28	***	−0.23	**
Acceptance	−0.41	***	−0.47	***	−0.29	***	−0.25	***	−0.36	***	−0.42	***	−0.43	***	−0.49	***
Religion	0.04		−0.04		0.02		0.003		0.13	*	0.04		0.14		0.14	
Behavioral disengagement	0.32	***	0.36	***	0.28	***	0.33	***	0.28	***	0.33	***	0.26	**	0.39	***
Self-distraction	0.15	***	0.01		0.13	*	0.14	**	0.25	***	−0.02		0.19	*	0.09	
Self-blame	0.51	***	0.41	***	0.40	***	0.33	***	0.48	***	0.36	***	0.48	***	0.48	***
Denial	0.27	***	0.26	***	0.21	*	0.14	**	0.31	***	0.26	***	0.37	***	0.30	***
Substance use	0.20	***	0.16	***	0.18		0.15	**	0.22	***	0.19	***	0.20	*	0.43	***

* *p* < 0.05, ** *p* < 0.01 and *** *p* < 0.001.

## References

[1] Cucinotta D., Vanelli M. (2020). WHO Declares COVID-19 a Pandemic. Acta Bio Med. Atenei Parm..

[2] Legifrance Décret n° 2020-260 du 16 Mars 2020 Portant Réglementation des Déplacements Dans le Cadre de la Lutte Contre la Propagation du Virus Covid-19. https://www.legifrance.gouv.fr/loda/id/JORFTEXT000041728476/.

[3] Legifrance Décret n° 2020-1310 du 29 Octobre 2020 Prescrivant les Mesures Générales Nécessaires Pour Faire Face à l’Épidémie de Covid-19 Dans le Cadre de l’État d’Urgence Sanitaire. https://www.legifrance.gouv.fr/jorf/id/JORFTEXT000042475143.

[4] Legifrance Décret n° 2020-1582 du 14 Décembre 2020 Modifiant les Décrets n° 2020-1262 du 16 Octobre 2020 et n° 2020-1310 du 29 Octobre 2020 Prescrivant les Mesures Générales Nécessaires Pour Faire Face à l’Épidémie de Covid-19 dans le Cadre de l’État d’Urgence Sanitaire. https://www.legifrance.gouv.fr/jorf/id/JORFTEXT000042665612.

[5] Ministère Français de lʼEnseignement Supérieur de la Recherche et de lʼInnovation Circulaire du 22 Janvier 2021 Portant sur l’Actualisation des Consignes Concernant la Reprise Progressive des Enseignements dans les Établissements de l’Enseignement Supérieur à Partir du 25 Janvier. https://services.dgesip.fr/fichiers/CirculaireRepriseEnseignements-22janvier21.pdf.

[6] Lee J. (2020). Mental health effects of school closures during COVID-19. Lancet Child Adolesc. Health.

[7] Sahu P. (2020). Closure of universities due to Coronavirus disease 2019 (COVID-19): Impact on education and mental health of students and academic staff. Cureus.

[8] Zhai Y., Du X. (2020). Addressing collegiate mental health amid COVID-19 pandemic. Psychiatry Res..

[9] Essadek A., Rabeyron T. (2020). Mental health of French students during the Covid-19 pandemic. J. Affect. Disord..

[10] Husky M.M., Kovess-Masfety V., Swendsen J.D. (2020). Stress and anxiety among university students in France during Covid-19 mandatory confinement. Compr. Psychiatry.

[11] Kaparounaki C.K., Patsali M.E., Mousa D.-P.V., Papadopoulou E.V., Papadopoulou K.K., Fountoulakis K.N. (2020). University students’ mental health amidst the COVID-19 quarantine in Greece. Psychiatry Res..

[12] Le Vigouroux S., Goncalves A., Charbonnier E. (2021). The psychological vulnerability of French university students to the COVID-19 confinement. Health Educ. Behav..

[13] Odriozola-González P., Planchuelo-Gómez A., Irurtia M.J., de Luis-García R. (2020). Psychological effects of the COVID-19 outbreak and lockdown among students and workers of a Spanish university. Psychiatry Res..

[14] Adalja A.A., Toner E., Inglesby T.V. (2020). Priorities for the US health community responding to COVID-19. JAMA.

[15] Yao H., Chen J.-H., Xu Y.-F. (2020). Patients with mental health disorders in the COVID-19 epidemic. Lancet Psychiatry.

[16] De Paula W., Breguez G.S., Machado E.L., Meireles A.L. (2020). Prevalence of anxiety, depression, and suicidal ideation symptoms among university students: A systematic review. Braz. J. Health Rev..

[17] Stanley J.M.N. (2001). Responding to students’ mental health needs: Impermeable systems and diverse users. J. Ment. Health.

[18] Costanza A., Di Marco S., Burroni M., Corasaniti F., Santinon P., Prelati M., Chytas V., Cedraschi C., Ambrosetti J. (2020). Meaning in life and demoralization: A mental-health reading perspective of suicidality in the time of COVID-19. Acta Bio Med. Atenei Parm..

[19] Wang C., Pan R., Wan X., Tan Y., Xu L., Ho C.S., Ho R.C. (2020). Immediate psychological responses and associated factors during the initial stage of the 2019 Coronavirus Disease (COVID-19) epidemic among the general population in China. Int. J. Environ. Res. Public Health.

[20] Cao W., Fang Z., Hou G., Han M., Xu X., Dong J., Zheng J. (2020). The psychological impact of the COVID-19 epidemic on college students in China. Psychiatry Res..

[21] Galea S., Merchant R.M., Lurie N. (2020). The mental health consequences of COVID-19 and physical distancing: The need for prevention and early intervention. Depress. Anxiety.

[22] Killgore W.D., Cloonan S.A., Taylor E.C., Dailey N.S. (2020). Loneliness: A signature mental health concern in the era of COVID-19. Psychiatry Res..

[23] Zhai Y., Du X. (2020). Mental health care for international Chinese students affected by the COVID-19 outbreak. Lancet Psychiatry.

[24] Réveillère C., Nandrino J.-L., Sailly F., Mercier C., Moreel V. (2001). Étude des tracas quotidiens des étudiants: Liens avec la santé perçue. Ann. Méd. Psychol. Rev. Psychiatr..

[25] Araújo F.J.D.O., De Lima L.S.A., Cidade P.I.M., Nobre C.B., Neto M.L.R. (2020). Impact of Sars-Cov-2 and its reverberation in global higher education and mental health. Psychiatry Res..

[26] Lazarus R.S., Folkman S., Gentry W.D. (1984). Coping and adaptation. The Handbook of Behavioral Medicine.

[27] Muller L., Spitz E. (2003). Évaluation multidimensionnelle du coping: Validation du Brief COPE sur une population française. Encephale.

[28] Dawson D.L., Golijani-Moghaddam N. (2020). COVID-19: Psychological flexibility, coping, mental health, and wellbeing in the UK during the pandemic. J. Context. Behav. Sci..

[29] Fiorillo A., Gorwood P. (2020). The consequences of the COVID-19 pandemic on mental health and implications for clinical practice. Eur. Psychiatry.

[30] Gunnell D., Appleby L., Arensman E., Hawton K., John A., Kapur N., Khan M., O’Connor R.C., Pirkis J., Caine E.D. (2020). Suicide risk and prevention during the COVID-19 pandemic. Lancet Psychiatry.

[31] Niederkrotenthaler T., Gunnell D., Arensman E., Pirkis J., Appleby L., Hawton K., John A., Kapur N., Khan M., O’Connor R.C. (2020). Suicide research, prevention, and COVID-19. Crisis.

[32] Lépine J., Godchau M., Brun P. (1985). Anxiety and depression in inpatients. Lancet.

[33] Ojewale L.Y. (2020). Psychological state and family functioning of University of Ibadan students during the COVID-19 lockdown. medRxiv.

[34] Boujut E., Koleck M., Bruchon-Schweitzer M., Bourgeois M.-L. (2009). La santé mentale chez les étudiants: Suivi d’une cohorte en première année d’université. Ann. Méd. Psychol. Rev. Psychiatr..

[35] Bonanno G.A., Ho S.M.Y., Chan J.C.K., Kwong R.S.Y., Cheung C.K.Y., Wong C.P.Y., Wong V.C.W. (2008). Psychological resilience and dysfunction among hospitalized survivors of the SARS epidemic in Hong Kong: A latent class approach. Health Psychol..

[36] Lee A.M., Wong J.G., McAlonan G.M., Cheung V., Cheung C., Sham P.C., Chu C.-M., Wong P.-C., Tsang K.W., Chua S.E. (2007). Stress and psychological distress among SARS Survivors 1 year after the outbreak. Can. J. Psychiatry.

[37] Liu X., Kakade M., Fuller C.J., Fan B., Fang Y., Kong J., Guan Z., Wu P. (2012). Depression after exposure to stressful events: Lessons learned from the severe acute respiratory syndrome epidemic. Compr. Psychiatry.

[38] Lang F.R., Carstensen L.L. (2002). Time counts: Future time perspective, goals, and social relationships. Psychol. Aging.

[39] Balayssac D., Pereira B., Darfeuille M., Cuq P., Vernhet L., Collin A., Vennat B., Authier N. (2018). Use of psychotropic medications and illegal drugs, and related consequences among French pharmacy students—SCEP study: A nationwide cross-sectional study. Front. Pharmacol..

[40] Baticulon R.E., Sy J.J., Alberto N.R.I., Baron M.B.C., Mabulay R.E.C., Rizada L.G.T., Tiu C.J.S., Clarion C.A., Reyes J.C.B. (2021). Barriers to online learning in the time of COVID-19: A national survey of medical students in the Philippines. Med. Sci. Educ..

[41] Peretti-Watel P., Seror V., Cortaredona S., Launay O., Raude J., Verger P., Beck F., Legleye S., L’Haridon O., Ward J. (2021). Attitudes about COVID-19 lockdown among general population, France, March 2020. Emerg. Infect. Dis..

[42] Satici B., Saricali M., Satici S.A., Griffiths M.D. (2020). Intolerance of uncertainty and mental wellbeing: Serial mediation by rumination and fear of COVID-19. Int. J. Ment. Health Addict..

[43] Garnefski N., Legerstee J., Kraaij V.V., Kommer T.V.D., Teerds J. (2002). Cognitive coping strategies and symptoms of depression and anxiety: A comparison between adolescents and adults. J. Adolesc..

[44] Whatley S.L., Foreman A.C., Richards S. (1998). The relationship of coping style to dysphoria, anxiety, and anger. Psychol. Rep..

[45] Le Vigouroux S., Dauvier B., Congard A., Kop J.-L., Pavani J.-B., Gilles P.-Y. (2015). Le développement des stratégies de régulation affective au cours de l’âge adulte. L’Année Psychol..

[46] Amerio A., Brambilla A., Morganti A., Aguglia A., Bianchi D., Santi F., Costantini L., Odone A., Costanza A., Signorelli C. (2020). COVID-19 Lockdown: Housing Built Environment’s Effects on Mental Health. Int. J. Environ. Res. Public Health.

[47] Flaudias V., Zerhouni O., Pereira B., Cherpitel C.J., Boudesseul J., de Chazeron I., Romo L., Guillaume S., Samalin L., Cabe J. (2021). The Early Impact of the COVID-19 Lockdown on Stress and Addictive Behaviors in an Alcohol-Consuming Student Population in France. Front. Psychiatry.

[48] Durrett C., Trull T.J. (2005). An evaluation of evaluative personality terms: A comparison of the Big Seven and Five-factor model in predicting psychopathology. Psychol. Assess..

[49] Kuppens P., Oravecz Z., Tuerlinckx F. (2010). Feelings change: Accounting for individual differences in the temporal dynamics of affect. J. Pers. Soc. Psychol..

[50] Widiger T.A., Trull T.J. (1992). Personality and psychopathology: An application of the five-factor model. J. Pers..

[51] Baertschi M., Costanza A., Canuto A., Weber K. (2018). The function of personality in suicidal ideation from the perspective of the interpersonal-psychological theory of suicide. Int. J. Environ. Res. Public Health.

